# Entrustable Professional Activities and Learning: The Postgraduate Trainee Perspective

**DOI:** 10.1007/s40596-022-01712-2

**Published:** 2022-10-12

**Authors:** Alice Stephan, Gary Cheung, Cees van der Vleuten

**Affiliations:** 1 Mental Health and Addictions Service, Waikato District Health Board, Hamilton, New Zealand; 2 School of Medicine, Faculty of Medical and Health Sciences, The University of Auckland, Auckland, New Zealand; 3 School of Health Professions Education, Faculty of Health, Medicine and Life Sciences, University of Maastricht, Maastricht, Netherlands

**Keywords:** Entrustable professional activities, Learning, Motivation, Faculty development, Postgraduate psychiatry training

## Abstract

**Objective:**

Entrustable professional activities (EPAs) are used as clinical activities in postgraduate psychiatry training in Australasia. This study aimed to explore psychiatry trainees’ perceptions of the impact of EPAs on their motivation and learning.

**Methods:**

A constructivist grounded theory approach was used to conceptualize the impact of EPAs on trainees’ motivation and learning. A purposive sample of trainees was recruited from across New Zealand. Semi-structured individual interviews were used for data collection and continued until theoretical saturation was reached.

**Results:**

The impact of EPAs on learning was mediated by the trainee’s appraisals of subjective control, value, and the costs of engaging with EPAs. When appraisals were positive, EPAs encouraged a focus on particular learning needs and structured learning with the supervisor. However, when appraisals were negative, EPAs encouraged a superficial approach to learning. Trainee appraisals and their subsequent impact on motivation and learning were most affected by EPA granularity, alignment of EPAs with clinical practice, and the supervisor’s conscientiousness in their approach to EPAs.

**Conclusions:**

To stimulate learning, EPAs must be valued by both trainees and supervisors as constituting a coherent work-based curriculum that encompasses the key fellowship competencies. If EPAs are to be effective as clinical tasks for learning, ongoing faculty development must be the leading priority.

Entrustable professional activities (EPAs) are used increasingly in postgraduate medical training, yet relatively little is known about their consequences on learning and professional practice. EPAs were developed as clinical tasks in teaching and learning to bridge the gap between the abstract notion of competencies and the practicalities of day-to-day clinical practice [1–4]. EPAs are authentic whole tasks, which can be performed, observed, and assessed in the workplace [2, 5]. An EPA is entrusted when a supervisor is satisfied that the trainee is competent to perform the task without direct supervision [2, 5]. The concept of entrustment formalizes the long existing practice of supervising medical specialists giving graded responsibility to trainees when they appear capable of managing a task safely [6].

EPAs are used to improve constructive alignment within a competency-based educational framework. EPAs have two key functions — to motivate learning and to assess competency [7, 8]. While the issues of EPA development, implementation, and entrustment have been well described in the literature [9], their impact on motivation and learning has not yet been studied. This knowledge is essential if EPAs are to be used positively to influence learning. A motivational framework could be used to explore the impact of EPAs on learning.

Motivation theories suggest that in achievement situations, cognitive appraisals, associated emotions, and the learning environment reciprocally interact to greatly influence motivation, engagement, and learning [10]. Cognitive appraisals of control, value, and cost have been recognized. Appraisals of subjective control relate to an individual’s expectancy of success. They include the perception of being able to initiate an action (action-control expectancy) and that this action will produce a positive outcome (action-outcome expectancy) [10, 11]. Value appraisals include intrinsic value, which relates to the subjective value of the activity, such as interest, enjoyment, and immediate relevance [8, 9], whereas utility value relates to the usefulness of the outcomes [10, 12]. Eccles and Wigfield (2002) also consider cost appraisals, which refer to an individual’s beliefs about the negative aspects of engaging in the achievement activity [12].

Positive control (anticipating success) with high intrinsic value appraisals (such as important, interesting, and relevant) encourages positive and activating emotions. These emotions usually stimulate motivation, commitment, and absorption in learning tasks. In turn, this promotes the use of flexible learning strategies and self-regulation strategies, which have positive impacts on learning and performance [10, 11, 13]. In contrast, a perception of limited control (anticipating failure) and low intrinsic value (such as unimportant, boring, and irrelevant) can induce negative and deactivating emotions. This lessens effort and information processing, thus restricting learning and reducing performance [10, 11, 13].

Contextual factors impact learning through their influence on achievement emotions and related appraisals [8]. However, the postgraduate clinical workplace, with a high demand for patient care, is known to be a highly challenging learning environment [5, 14, 15]. Numerous studies have highlighted problems with teaching in this context, including infrequent direct observation of trainee practice and poor feedback from supervisors [14, 16]. Workplace assessments have been widely introduced with the intention of improving feedback for learning; however, they have not been an unqualified success. Motivating and engaging trainees and supervisors in meaningful workplace assessment for learning has been problematic [7, 15, 17, 18]. EPAs are increasingly adopted in postgraduate medical education and introducing EPAs typically leads to an increased number of workplace-based assessments. Therefore, it is crucial that we obtain a better understanding of the consequences of EPAs for learning. In this study, a constructivist grounded theory approach was utilized to explore trainees’ perceptions of how EPAs impacted their motivation and learning.

## Methods

Postgraduate specialist psychiatry training in Australia and New Zealand is offered through the Royal Australia New Zealand College of Psychiatrists (RANZCP). The 5-year fellowship program consists of a series of six-monthly clinical placements working alongside a psychiatrist supervisor to complete: stage 1 (12 months fulltime equivalent [FTE]), stage 2 (24 months FTE), and stage 3 (24 months FTE). RANZCP training uses the CanMEDS competency framework with EPAs as central work-based summative assessments. An EPA is entrusted when a trainee’s supervisor is confident that the required clinical competence has been demonstrated to the specified standard. The individual supervisor’s entrustment decision is made on the basis of all available data, which must be informed by a minimum of three formative work-based assessments [3]. The fellowship program uses five standardized formative work-based assessment tools: case-based discussion, mini-clinical evaluation exercise, professional presentation, observed clinical interview, and direct observation of procedure [3]. Progression in RANZCP fellowship training requires that trainees have EPAs accredited for each rotation. Depending on the specific clinical placements, a trainee will be required to have between 25 and 30 EPAs entrusted for RANZCP fellowship. On average, trainees have six EPAs entrusted by their supervisor annually throughout their first 3 years of training, the bulk of which are usually entrusted in stage 2 during the second and third years of training [3].

A constructivist grounded theory approach was used to conceptualize how EPAs might impact postgraduate trainees’ motivation and learning. The constructivist paradigm recognizes that both data and interpretations are jointly constructed through interactions between participants and researchers [19, 20]. While the viewpoint of the trainees is central, the vantage point of the researchers is also acknowledged [20, 21]. The research team was composed of a psychiatrist and director of training of one of the five New Zealand RANZCP psychiatric training programs, an academic psychiatrist, and a professor of education with expertise in assessment. The researchers’ experiences in medical education and as psychiatrists informed their choice of theoretical framework as well as the development of the research questions, the interview pro-forma, data analysis, and interpretation.

A purposive sample of stage 2 and 3 RANZCP trainees was recruited from five New Zealand training sites in 2017. Following ethics approval, the study was introduced to a combined meeting of the New Zealand directors of training by the lead researcher, who also followed up with each director of training individually. Each director of training invited trainees from their local training scheme to participate. Invitations varied from a forwarded email participant information sheet and the standard invitation to participate, to intentional targeting of individual trainees, who were thought likely to provide “rich information” [20], for example, a trainee who might give a longitudinal perspective by virtue of being near the end of training, or have a broader perspective because of their involvement with the RANZCP, or have an alternative viewpoint due to previous postgraduate training. If a trainee wished to participate in this study, they contacted the lead researcher. Trainees from across all five New Zealand training sites were represented in the study.

Individual interviews were chosen for data collection because internal psychological processes related to motivation and learning were the primary area of interest. Interview questions were developed to encourage interviewees to recount and reflect upon specific personal experiences with EPAs. Semi-structured interviews varied between 45 and 60 min in length. Open-ended questions encouraged interviewees to recount specific experiences with EPAs and their associated reflections and perspectives on factors that might have influenced their experiences with EPAs. They aimed to elicit data on different domains of experiences including feelings, beliefs, behavior, and opinions. For example, how are you finding the EPAs? What are they like for you in practice? Can you tell me about a specific EPA, or an event related to an EPA that stands out clearly in your mind? Consistent with the principle that the theory arises from the data [20], questioning was not guided by any theoretical concepts including motivation and achievement theories [10, 12]. For ethical reasons and to minimize power imbalance and conflict of interest, researchers did not interview trainee participants in their training program.

Narratives were recorded and transcribed verbatim and the transcripts subsequently entered into NVivo, a computer-assisted qualitative data analysis program. The researchers engaged in an iterative process with data collection and data analysis alternating, meaning transcripts and ideas from earlier interviews influenced subsequent data collection and interpretation [20]. Early transcripts were read and then coded independently by the first and second author, who agreed on initial categories which focused on the text in detail, such as phrases or sentences to cover the scope of the data. In a process of constant comparison working from the detailed to more general, the features of each category were elucidated and refined [20, 21]. Exceptions were particularly considered to ensure they could be accounted for in the categorization. The data was repeatedly reread in its entirety and integrated with the categories to arrive at broader themes or core categories [20, 21]. Inter-relationships between themes were explored generating a series of hypotheses which were represented in both narrative and diagrammatic forms. Throughout this process, the researchers attempted to keep an open-minded and non-theoretical approach [20].

The researchers then reconsidered the data in the light of motivation theories [10, 12]. Watling and Lingard [20] suggest that “existing theoretical frameworks might complement or extend the data interpretation or offer alternate explanations for challenging data” (p. 858). The lead researcher repeated the previously outlined process, noting examples that were consistent with these theoretical constructs, while not discounting counter-examples. Where applicable, concepts from motivation theories were used in analysis of the results and in the theoretical model that emerged. Data collection continued through theoretical sampling until saturation was reached, that is, sufficient data to support the development of a plausible model linking EPAs, motivation, and learning [20, 21].

## Results

A total of 12 trainees (equal numbers of males and females) participated in this study. Four trainees were in years 2 to 3 of training (stage 2), four trainees had just begun year 4 (stage 3), and four were in their final year of training.

**Fig. 1:**
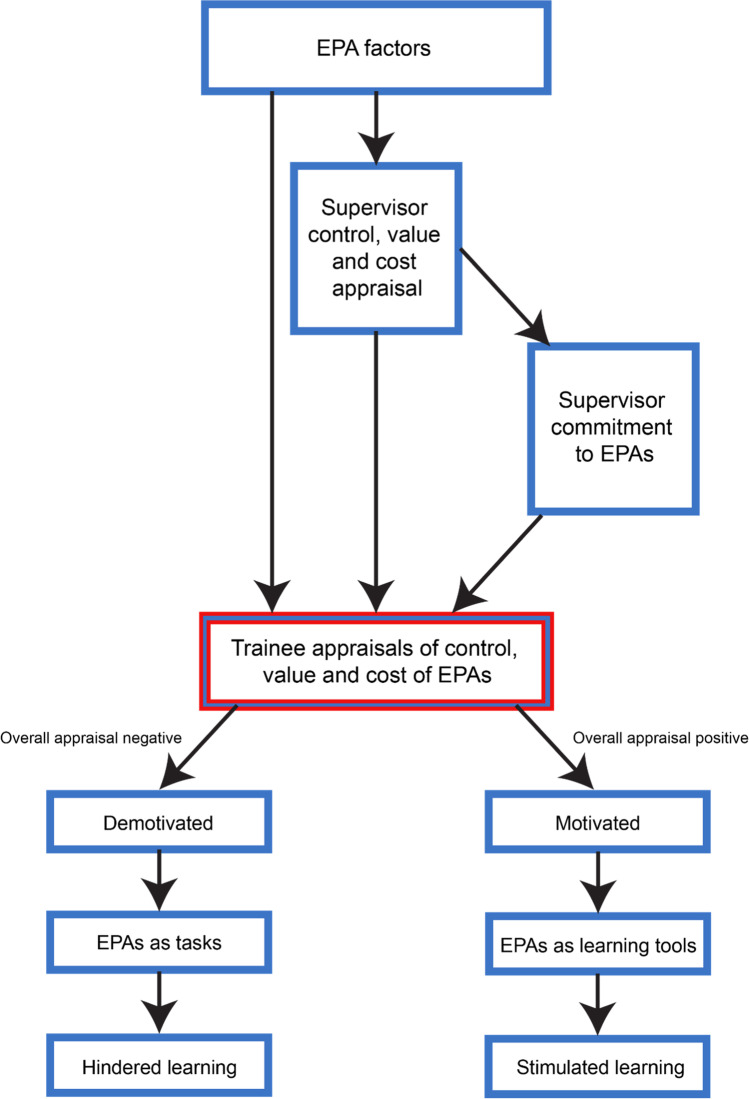
Trainee perceptions of the impact of entrustable professional activities (EPAs) on their motivation and learning

The data analysis indicated that the impact of EPAs on trainee learning was variable; EPAs stimulated learning in some instances, but hindered learning in others. A model was developed whereby trainee appraisals were central in determining their level of motivation and engagement with EPAs. When appraisals of value and control were high and cost appraisals were low, EPAs positively impacted learning. Conversely, when appraised unfavorably, a more superficial approach to learning resulted. Factors that influenced the trainee’s appraisals and subsequent motivation to engage with the EPAs, related to the EPAs themselves as well as the supervisor’s level of commitment to using EPAs as a learning tool (Fig. 1).

### EPAs Stimulated Learning

When appraised positively, EPAs were appreciated for providing a structured approach to learning, and were generally associated with positive and activating emotions such as exciting, interesting, and enjoyable.

Early in training, EPAs were often used to guide value appraisals, which assisted with focusing and prioritizing trainee learning. For example, one trainee commented:

“I’ve got to do all these EPAs … I got excited … in that way it was good for me because I didn’t know, I was new to it… and it was quite nice to have a directive sort of way of learning.” (Participant 1)

EPAs contributed to a positive expectancy that the trainee had control over the learning process and that a successful outcome would ensue:

“When I did the Mental Health Act EPA it was good because … you knew what you would have to do... you have to go to Mental Health Act Court, you have to write a Mental Health Act report, and then obviously just go through the Mental Health Act process … and I could direct that myself as well … it probably felt like I was more in control of that.*”* (Participant 1)

In some instances, EPAs motivated and empowered trainees to expand from the more routine aspects of their role to focus on their particular training needs or to create specific learning opportunities:

“The supportive psychotherapy EPA, it made me approach [my supervisor] saying, ‘I need patients for this’, and my current supervisor is very good, so she keeps recommending me people.” (Participant 3)

Some trainees used EPAs as leverage to encourage input from more reluctant supervisors:

“Having the guidance what you are expected to know, I think it is actually pretty helpful, and particularly if you have supervisors who would otherwise be a bit ‘hands off’, you can say obviously, they have to get the stuff done so we have to talk about it.” (Participant 4)

EPAs offer a structure for learning. Each EPA descriptor outlines the clinical task, with expected competencies specified in terms of knowledge, attitude, and skills. Each decision about entrustment must be informed by at least three work-based assessments. Suggestions about appropriate combinations of different work-based assessments types are given. This structured learning process resulted in a more thorough approach to learning with their supervisor than was usually the case when they were not engaged in an EPA. One trainee described the case-based discussion process:

“There would be a lot more rigorous questioning around the patient, and the management of the patient, rather than me just very briefly giving an overview … would I usually have those discussions even if there wasn’t an EPA? No, because of time constraints. I don’t recall having a conversation about a patient for 45 minutes … without being in the context of a work-based assessment.” (Participant 5)

Direct observation of practice was particularly appreciated. Trainees reported that when done well, EPAs encouraged feedback-type conversations with supervisors, which assisted with goal setting and retaining a joint focus on areas purposefully targeted for improvement.

When well supported by the supervisor and the wider multidisciplinary team, EPAs broadened and deepened learning. The following example illustrates how garnering support positively influenced a trainee’s appraisals of control (achievable and realistic) and value (important and useful), thus, motivating and fostering engagement, with deeper learning ensuing:

“The Māori formulation case, I guess that was interesting because we had some discussion with a Māori mental health worker. I did some reading and talked about it in more depth and I’m not sure we would have necessarily done that as much if I hadn’t been doing that EPA. So that was important and useful, good … motivating, because we discussed the EPA beforehand, worked out whether it was achievable and realistic for that run and then we planned out what would work, and what’s appropriate, and to think about it and what I could do … I think it enhanced my interest in that topic.” (Participant 7)

### EPAs Hindered Learning

Alternatively, as training progressed, EPAs tended to be appraised as having limited value, to be associated with negative emotions and a more superficial approach to learning:

“They take up a lot of time … [I am] mostly frustrated by them and don’t necessarily find them helpful.” (Participant 8)

“EPAs are a series of hoops that we have to jump through in order to get fellowship. There is a sense that it diminishes the joy or excitement of learning in psychiatry.” (Participant 6)

Accompanying appraisals of low intrinsic value were perceptions of significant opportunity costs. Several trainees recounted how they missed out on other potentially more worthwhile or personally necessary learning opportunities due to EPA requirements. EPAs were experienced as dominating supervision; limiting or “pushing out” other beneficial interactions with their supervisor, such as mentoring or reflecting on personal challenges or difficult cases; and in some instances, to the detriment of trainee wellbeing:

“You also want to discuss the stuff that is not core RANZCP but it’s important for your career and for your mental health.” (Participant 4)

It was generally considered that the EPA structure made it difficult to follow one’s own interests however relevant:

“There are things that would have been really interesting to me that I haven’t done for that reason because there is such a burden of learning that we have to sit this quite rigid structure, but there is not a lot of flexibility for pursuing other interests … you avoid things that won’t count towards your training.” (Participant 6)

Trainees appraised EPAs as diminishing their subjective control of their own learning and linked this with diminished enjoyment of learning:

“Learning at its best is enjoyable isn’t it, and if you make it too rigid, too concrete then it reduces how enjoyable it is, and so you get less out of it and then it becomes a bit more of a dreaded thing …” (Participant 9)

### External Influencing Variables

The main external variables that influenced trainee learning were EPA granularity and the degree of supervisor commitment to EPAs. The impact of these variables was mediated through subjective control, value, and cost appraisals, which guided trainee motivation and engagement.

#### EPA Granularity

The granular nature of the EPAs led to issues about EPA topics, the overall scope of EPAs, and EPA numbers. Trainees universally thought the requirement to have 25 to 30 EPAs entrusted for RANZCP Fellowship was excessive. During stages 1 and 3 when there were fewer EPAs, trainees generally appraised EPAs more positively and reported better learning. In stage 2, with more pressure for EPA entrustment, value appraisals and control appraisals became progressively more negative, while the costs compounded as did negatively valanced emotions:

“Looking at the list of EPAs … it’s pretty intimidating, it’s pretty exhausting … most trainees at the beginning of Stage 2 have the experience of feeling quite overwhelmed …” (Participant 10)

A disengaged learning style with more superficial learning was described:

“So, you just want to get them out of the way and that to my mind is the motivation. You’ve got to get them done so you will get them done, rather than get them done to learn.” (Participant 11)

The appraisal of any single EPA was influenced by a number of different factors including personal priorities, priorities of the supervisor, and the service:

“If it melds well with what is the bread and butter of a particular subspecialty or particular rotation … it forms a good backbone, but … when they have very little to do with the actual rotation that you are doing … you end up seeing it as a little bit of a left field burden and you just try and quickly scatter and complete it.” (Participant 12)

As training progressed, trainees became more critical about the scope of the EPAs, which were seen as somewhat haphazardly put together and not necessarily reflecting the fellowship competencies holistically:

“It also seems a bit arbitrary to what is deemed to be an important competency and what is not.” (Participant 12)

There was however a sense that this was unsatisfactory:

“At the end of your training there should be core things you can do ... more clearly related to the day-to-day job … those are the things that should be tested in EPAs … EPAs need to broadly reflect the core competencies of a psychiatrist.” (Participant 8)

#### Supervisor Commitment to EPAs

For most (but not all) trainees, their appraisals of the EPAs appeared to be strongly influenced by their perception of their supervisor’s attitude towards the EPAs. The supervisor’s attitude along with their commitment to using EPAs as a learning tool was identified by the participants as crucial, if EPAs were to be beneficial for their learning:

“The usefulness depends on the supervisor you have and how trained they are in using EPAs, not just trained but also how much they believe in the usefulness of EPAs.” (Participant 5)

Discrepancies between supervisors in this regard were evident along a number of dimensions, including their use of EPAs as assessment for learning:

“It is incredibly variable as to how thoroughly one’s consultant uses this as a tool for helping you learn the right skills or work through the right knowledge base; from complete tokenism, as in this case vaguely looks like it might fit under that heading; through to actually, let’s make sure you know all the components as written down in the EPAs that we’ve covered during our discussions as we’ve gone through various patients”. (Participant 2)

Similarly, the rigorousness of entrustment decisions varied substantially with supervisor commitment. The majority of supervisors were perceived to be only superficially acquainted with EPA requirements:

“We will run through with what’s vaguely expected but I would be very surprised if they have much of an overview of what’s involved and the overall RANZCP expectations”. (Participant 4)

Generally, trainees thought their learning would improve if entrustment decisions were informed by more direct observation of practice. Lax interpretations of the EPA competency standards by some supervisors were seen as potentially undermining EPA credibility:

“The other variable is the level they are assessed at. I guess, most obviously in Stage 1 where you can do some Stage 2 EPAs, and your supervisor will tick you off in your first run of Stage 1, for something that you in theory, are doing at the proficiency of somewhere at the end of year three, it doesn’t quite seem right”. (Participant 8)

Systemic issues such as lack of support and training for supervisors were identified as impacting on the supervisors’ abilities to use EPAs as educational tools. Trainees thought that the supervisors were largely under-prepared for their role as educators:

“Because the consultants haven’t been trained, they don’t know what they are doing.” (Participant 2)

Several trainees alluded to workplace pressure as making it difficult for supervisors to adequately engage with the EPAs as clinical tasks for learning:

“I think in theory they are good, but in a practical sense it’s just logistically challenging ... if the service is so busy that you [the supervisor] can’t come and sit in with your registrar, it just kind of seems like a token.” (Participant 2)

“It only works if the whole system is teaching and that is where it falls down”. (Participant 2)

## Discussion

In contrast to the poor reception and implementation of work-based assessments generally [14, 15, 17, 18], there has been optimism that EPAs may advance assessment for learning in postgraduate medical education [7]. This study indicated that the impact of EPAs on learning was mediated by the trainees’ appraisals of subjective control, value, and the costs of engaging with EPAs. Their appraisals were most effected by EPA characteristics such as EPA granularity and perceptions of clinical relevance, as well as the supervisor’s conscientiousness in their approach to EPAs.

Turning attention first to the EPAs themselves, entrustment of EPAs was a requirement for progression in training. When their numbers were modest, EPAs were more likely to be educationally positive. As numbers increased the costs compounded, appraisals of intrinsic value became progressively more negative, as did appraisals of subjective control of learning. This was associated with lessening of motivation, diminished enjoyment, and more superficial learning. Trainees uniformly considered that the number of EPAs (25 to 30 over 5 years) undermined successful assessment for learning. This is consistent with a previous survey where RANZCP trainees have reported the burden of work-based assessments as excessive [22]. Initial recommendations for a full postgraduate training program were 20 to 30 EPAs [23], but recently, significantly fewer EPAs have been suggested. For example, 13 end-of-training EPAs are proposed for graduating psychiatrists in Canada [24], whereas in the Netherlands, the curricular working group for psychiatry has preliminarily identified eight essential EPAs [25].

Trainee appraisals of EPAs tended to more positive earlier in training, when expectations that EPAs defined a cohesive curriculum led to positive value appraisals and structured learning supported positive control appraisals. As training progressed however, EPAs were more often seen as creating learning opportunities at the cost of personal agency and enjoyment, while constraining other potentially more valuable learning opportunities.

In addition to the pressure of numbers, there were issues relating to the scope and complexity of EPAs. Trainees did not always consider that EPAs aligned well with clinical priorities and were skeptical whether they captured the essential competencies of a psychiatrist. These ideas are taken up in the literature, which recommends a tight alignment of EPAs with clinical practice. The underlying construct is that an EPA must constitute a meaningful and accurate description of the essential work required for professional practice [26, 27]. This applies both to individual EPAs and to the set of EPAs that form the workplace curriculum. Mulder, ten Cate, Daalder, and Berkvens (2010) stated, “the set of EPAs identified when building a workplace curriculum should be a valid coverage of the profession and all domains of competence should achieve attention in a well-balanced way” [26: 454]. The authors go on to say that when building a curriculum, the scope and complexity of EPAs, as well as the tension between granularity and numbers needs to be considered.

Developing a set of EPAs that deliver valid descriptions of professional practice can be challenging. Taylor et al. (2021) suggest that using construct validity to evaluate each stage of the EPA development process would improve confidence that EPAs are appropriate for workplace use [27]. They suggest that all processes should be scrutinized through the “construct validity” lens. This would include the selection of expert panel members; identification of potential EPAs, the EPA descriptors, and iterative reviews of EPAs; and adoption of EPAs. EPAs for the RANZCP curriculum were selected on the basis of importance in daily practice, high-risk or error-proneness, and relevance to multiple major CanMEDS roles [3]. They were initially developed by a combination of expert consensus and composite working parties of relevant stakeholders [28]. As EPAs were only one element in a comprehensive redesign of the RANZCP fellowship program, their development occurred stepwise such that stage 1 was being implemented before the stage 2 EPAs were finalized. Likewise, stage 2 was implemented before stage 3 EPAs were completed [3]. As a consequence, individual EPAs vary in terms of breadth, granularity, and complexity. It seems unlikely that the core stage 1 and 2 EPAs if combined would form a cohesive whole, the scope of which would outline a comprehensive and coherent work-based curriculum.

Turning now to the impact of the supervisor on the trainee’s appraisals of EPAs, motivation, and learning, it seemed the supervisor also appraised the value, feasibility, and opportunity costs of the EPAs and these appraisals appeared to be pivotal in the supervisor’s subsequent level of engagement with the EPAs. It appeared that the supervisor’s appraisals and engagement influenced the trainee’s evaluations and level of commitment to using the EPAs for learning. The finding that the supervisor’s conscientiousness largely determined the usefulness of EPAs for trainee learning resonated with other published literature [15, 29]. The participating trainees echoed the comments of other RANZCP trainees that there was considerable variability between supervisors, dependent upon their interest in training [22] and their commitment to the use of EPAs. For the assiduous supervisor, EPAs were a tool which facilitated feedback on observed practice and in-depth probing of clinical reasoning and management, both key tasks of assessment for learning [18]. However, the call from trainees for higher quality feedback and more direct observation of practice was strong. This echoes a previous survey of RANZCP trainees [22] and the results of other studies on work-based assessments that identified insufficient direct observation [7, 15, 17].

Another variable impacting the appraisals of both trainees and supervisors with regard to implementation of EPAs was the value and support for training at the organizational level. Both RANZCP supervisors [30] and trainees [22] had previously identified a workplace culture that struggled to support competency-based training. Tension between increasingly stringent training requirements and growing clinical demands, in the context of insufficient resources and time, were key issues. This reflected the well-known difficulties of postgraduate training, which is typically situated in busy clinical environments with a high demand for patient care [7, 14, 15].

As a corollary, the lack of attention to ongoing faculty development for supervisors has been previously recognized [31] and was highlighted again by trainees in this study. It is well accepted that the chief impediment to successful implementation of competency-based medical education is a lack of realistically resourced and ongoing faculty development [7, 17, 18, 31, 32]. Consistent on-the-job training, rather than initial workshops as provided by RANZCP, is thought to be necessary for supervisors to reach and maintain the required level of expertise to implement competency-based training [17].

There are a number of limitations to be acknowledged. First, the use of individual interviews for data collection focused on the internal processes of trainees in relation to the judgments that affected their motivation and learning with EPAs. However, this individual focus is also a limitation because while it alludes to external influences, it only briefly touches on the work-based learning environment. It fails to explore the views of significant others such as supervisors, training directors, or clinical services. The supervisors’ and training directors’ perspectives on how to facilitate assessment for learning with EPAs warrant further research. Second, the participants were all training in psychiatry in New Zealand which may limit transferability of the findings to other contexts. Our sample size was also relatively small. Repeating this study elsewhere, with a larger number of psychiatry trainees in Australia and New Zealand and potentially with post-graduate trainees from other post-graduate training programs structured around EPAs, would indicate the degree to which these results are generalizable. Third, although choosing participants most likely to give “rich information” is consistent with the grounded theory principle of theoretical sampling, this recruitment process could have led to selection bias.

To conclude, as RANZCP was one of the first colleges to implement a postgraduate curriculum based on EPAs in 2012, it would seem appropriate to reappraise and rework not only the EPAs, but more importantly, their implementation. Broadening the scope of EPAs to facilitate reduction in the number of EPAs in line with numbers suggested for other postgraduate psychiatric curriculums is a priority. To stimulate learning, the sum of EPAs must describe a coherent work-based curriculum that encompasses the key fellowship competencies. This study highlighted that without skilled and well-supported faculty, the optimism that EPAs are advancing assessment for learning is misplaced.

## References

[1] ten Cate O (2013). Competency-based education, entrustable professional activities, and the power of language. J Grad Med Educ.

[2] ten Cate O, Snell L, Carraccio C (2010). Medical competence: the interplay between individual ability and the health care environment. Med Teach.

[3] Royal Australian and New Zealand College of Psychiatrists. EPA handbook stage 1 and 2. 2012. https://www.ranzcp.org/epahandbook.aspx. Accessed 25 Apr 2019.

[4] Pinilla S, Lenouvel E, Strik W, Klöppel S, Nissen C, Huwendiek S (2020). Entrustable professional activities in psychiatry: a systematic review. Acad Psychiatry.

[5] ten Cate O, Scheele F (2007). Competency-based postgraduate training: can we bridge the gap between theory and clinical practice?. Acad Med.

[6] Englander R, Carraccio C (2014). From theory to practice: making entrustable professional activities come to life in the context of milestones. Acad Med.

[7] Driessen E, Scheele F (2013). What is wrong with assessment in postgraduate training? Lessons from clinical practice and educational research. Med Teach.

[8] Lockyear J, Carraccio C, Chan M, Hart D, Smee S, Touchie C (2017). Core principles of assessment in competency based medical education. Med Teach.

[9] O’Dowd E, Lydon S, O’Connor P, Madden C, Byrne D (2019). A systematic review of 7 years of research on entrustable professional activities in graduate medical education, 2011–2018. Med Educ.

[10] Pekrun R (2006). The control-value theory of achievement emotions: assumptions, corollaries, and implications for educational research and practice. Educ Psychol Rev.

[11] Pekrun R, Goetz T, Frenzel A, Barchfeld P, Perry R (2011). Measuring emotions in students’ learning and performance: the achievement emotions questionnaire (AEQ). Contemp Educ Psychol.

[12] Eccles J, Wigfield A (2002). Motivational beliefs, values, and goals. Annu Rev Psychol.

[13] Huang C (2011). Achievement goals and achievement emotions: a meta-analysis. Educ Psychol Rev.

[14] Norcini J, Burch V (2007). Workplace-based assessment as an educational tool: AMEE guide no. 31. Med Teach.

[15] Dijksterhuis MGK, Schuwirth LWT, Braat DDM, Teunissen PW, Scheele F (2013). A qualitative study on trainees’ and supervisors’ perceptions of assessment for learning in postgraduate medical education. Med Teach.

[16] Holmboe E (2004). Faculty and the observation of trainees’ clinical skills: problems and opportunities. Acad Med.

[17] Bok H, Teunissen P, Favier R, Rietbroek N, Theyse L, Brommer H (2013). Programmatic assessment of competency-based workplace learning: when theory meets practice. BMC Med Educ.

[18] Holmboe E, Ward D, Reznick R, Katsufrakis P, Leslie K, Patel V (2011). Faculty development in assessment: the missing link in competency-based medical education. Acad Med.

[19] Bunniss S, Kelly DR (2010). Research paradigms in medical education research. Med Educ.

[20] Watling CJ, Lingard L (2012). Grounded theory in medical education research: AMEE guide no. 70. Med Teach.

[21] Creswell J (2012). Educational research: planning, conducting and evaluating quantitative and qualitative research.

[22] Stephan A, Cheung G (2017). Clinical teaching and supervision in postgraduate psychiatry training: the trainee perspective. Australas Psychiatry.

[23] ten Cate O (2014). AM last page: what entrustable professional activities add to a competency-based curriculum. Acad Med.

[24] Young J, Hasser C, Hung E, Kusz M, O’Sullivan P, Stewart C (2018). Developing end-of-training entrustable professional activities for psychiatry: results and methodological lessons. Acad Med.

[25] Rühl N, van Hulst BM, de Leede B, Oosterling A (2019). Entrustable professional for a curricular framework for psychiatry in the Netherlands. Tijdschr Psychiatr.

[26] Mulder H, ten Cate O, Daalder R, Berkvens J (2010). Building a competency-based workplace curriculum around entrustable professional activities: the case of physician assistant training. Med Teach.

[27] Taylor D, Park YS, Smith C, Cate OT, Tekian A (2021). Constructing approaches to entrustable professional activity development that deliver valid descriptions of professional practice. Teach Learn Med.

[28] Boyce P, Spratt C, Davies M, McEvoy P (2011). Using entrustable professional activities to guide curriculum development in psychiatry training. BMC Med Educ.

[29] van der Vleuten CPM, Schuwirth LWT, Scheele F, Driessen EW, Hodges B (2010). The assessment of professional competence: building blocks for theory development. Best Pract Res Clin Obstet Gynaecol.

[30] Cheung G, Stephan A (2017). Supervision: ‘a random bag of arrangements’? Perspectives from psychiatrists on how to improve clinical teaching. Australas Psychiatry.

[31] Carraccio CL, Englander R (2013). From Flexner to competencies: reflections on a decade and the journey ahead. Acad Med.

[32] Lupi C, Ownby A, Jokela J, Cutrer W, Thompson-Busch A, Catallozzi M (2018). Faculty development revisited: a systems-based view of stakeholder development to meet the demands of entrustable professional activity implementation. Acad Med.

